# Anaesthesia administered as S(+)-ketamine for cardiac intervention in children with common congenital heart disease

**DOI:** 10.1097/MD.0000000000031624

**Published:** 2022-11-04

**Authors:** Wenjun Liu, Fei Xu, Liyong Guo, Hong Li, Liyun Zhao, Jun Ma

**Affiliations:** a Department of Anesthesiology, Beijing Anzhen Hospital, Capital Medical University, Beijing 100029, China.

**Keywords:** anesthesia, cardiac intervention, children, congenital heart disease (CHD), S(+)-ketamine(Esketamine)

## Abstract

Safety and efficacy were assessed of different S(+)-ketamine doses combined with propofol administered as anesthesia to common pediatric congenital heart disease (CHD) patients undergoing cardiac interventional surgery to provide reference data as guidance for use in clinical settings.

**Methods:**

Sixty CHD children admitted to Beijing Anzhen Hospital, Capital Medical University from December 2020 to December 2021 who underwent elective cardiac intervention were assigned to 3 groups (H, L, M, 20 patients/group) using a random number table-based method. Patients received 1% propofol (2 mg/kg) and intravenous injections of S(+)-ketamine (Group L, 0.4 mg/kg; Group M, 0.5 mg/kg; Group H, 0.6 mg/kg) followed by intravenous pumping of 1% propofol (4–6 mg/kg/h). Heart rate (HR), mean arterial pressure, and pulse oxygen saturation were recorded preoperatively (T0), at the time of anesthesia maintenance (T1), at the time of arteriovenous puncture (T2), and when they awakened (T3). Additionally, propofol dose and incidence rates of intraoperative body movement, postoperative agitation, and postoperative nausea/vomiting were recorded.

**Results:**

For the 3 groups, Group H awakening time was significantly longer than that of Group L (*P* = .039). Notably, intergroup intraoperative propofol times differed significantly (*P* = .009). Meanwhile, T0 to T3 intragroup HR values differences were significant (*P* = .017; *P* = .001; *P* = .005, respectively). Group L HR was significantly elevated at T2 relative to T0 (*P* = .003), Group M HR was significantly elevated at T1 and T2 relative to T0 (*P* = .019; *P* = .003, respectively), and Group H HR values were significantly elevated at T1 and T2 relative to T0 (*P* = .012; *P* = .005, respectively). At all 4 time points no statistically significant intergroup differences in mean arterial pressure values were observed (*P* = .587). T1 to T3 pulse oxygen saturation values for all 3 groups were significantly greater than corresponding T0 values. Although intergroup intraoperative body movement incidence differed significantly (*P* = .044), intergroup differences in awakening time agitation and postoperative nausea/vomiting incidence rates were insignificant (*P* = .732, *P* = .887, respectively).

**Conclusion:**

Use of 0.6 mg/kg S(+)-ketamine with propofol was most effective as anesthesia for common pediatric CHD patients undergoing cardiac interventional surgery.

## 1. Introduction

Congenital heart disease (CHD) in children is relatively common in clinical practice and is caused mainly by atrial septal defects, ventricular septal defects, arteriovenous ductus arteriosus, and other diseases. In general, pediatric CHD is treated using cardiac interventional surgery, such as percutaneous interventional atrial septal defect closure, percutaneous interventional ventricular septal defect closure, percutaneous interventional patent ductus arteriosus closure, although such operations pose risks to pediatric patients due to their young ages, complexities of their CHD conditions, surgical difficulties, and the need for careful administration of anesthesia or general anesthesia to uncooperative patients or those with low pain tolerance. In order to ensure successful completion of arteriovenous puncture and stabilization of vital signs, it is essential to maintain appropriate depth of anesthesia. In clinical settings, anesthesiologists often use a combination of propofol and ketamine that offers several advantages, such as rapid onset, good effect, a high level of safety, and rapid awakening. S(+)-ketamine (esketamine) is a spin isomer of ketamine that has high potency with fewer adverse effects as compared with ketamine.^[1,2]^ However, use of combinations of S(+)-ketamine and propofol in clinical settings have not frequently been reported in China, prompting this study. Here, safety and efficacy were assessed of different doses of S(+)-ketamine administered with propofol during surgical cardiac interventions conducted in pediatric CHD patients in China.

## 2. Methods

### 2.1. Study design and participants

This study was a randomized controlled prospective trial of 60 patients with pediatric CHD who were admitted to Beijing Anzhen Hospital, Capital Medical University from December 2020 to December 2021. All patients underwent elective cardiac interventions to treat heart disorders that included ventricular septal defect (28 cases), atrial septal defect (27 cases), and patent ductus arteriosus (5 cases). The patient cohort included 32 males and 28 females belonging to cardiac function (New York Heart Association) classes I-II.

Inclusion criteria: Patients requiring cardiac intervention under general anesthesia; Patients of 1 to 5 years of age; Patients belonging to American Society of Anaesthesiologists classes II to III; and Patients with body masses within the range of 5.9 to 18.1 kg.

Exclusion criteria: Patients with additional neurological and psychiatric diseases; Patients with other organic diseases such as liver and kidney failure, increased intracranial pressure, and history of ocular hypertension; Patients with allergies to anesthetic drugs used in the study; Patients with histories of long-term or frequent use of analgesic, sedative, or psychotropic drugs; and Patients with active gastroaesophageal reflux disease.

Dropped out criteria: Patient withdrawal from the trial; Premature termination of the operation; Incidents of serious cardiopulmonary adverse events; and Failure of the patient to provide complete data after the operation.

Preoperatively all patients were required to fast for 6 hours and abstain from consuming liquids 2 hours prior to surgery. Peripheral venous access was established without preoperative administration of drugs before each patient entered the catheterization room. After entering the catheterization room, each patient received 1% propofol administered at a dose of 2 mg/kg (Beijing Fresenius Kabi Pharmaceutical Co., Ltd., Beijing Fresenius Kabi Pharmaceutical Company Limited ,Beijing city, China, product lot no. 10PK7764) in combination with atropine (0.01 mg/kg) via intravenous injection. After the patient fell asleep, a test dose of S(+)-ketamine (esketamine, manufacturer: Jiangsu Hengrui Pharmaceutical Co., Ltd., Jiangsu Hengrui Pharmaceuticals Co., Ltd. Lianyungang City, Jiangsu Province ,China, specification 2 mL:50 mg/stem, product lot no. 210225BL) was administered according to the test protocol followed by continuous intravenous pumping of 1% propofol (4–6 mg/kg/h). Next, the patient was placed in a supine position with shoulders elevated so that his/her head was tilted back. Spontaneous breathing was maintained by ensuring the entire airway was open while oxygen was administered using a nasal cannula (3 L/min). Patients received 8 to 10 mL/(kg-h) of glucose liquid electrolyte solution intravenously as appropriate. A multifunctional monitor was used to routinely monitor the patient’s status based on five-lead ECG, fingertip pulse oxygen saturation (SpO_2_), noninvasive arterial blood pressure, and heart rate (HR) readings.

Patients were randomly assigned to three groups (20 patients/group) using a random number table-based method and received slow intravenous injections of different doses of S(+)-ketamine (Group L, 0.4 mg/kg; Group M, 0.5 mg/kg; Group H patients, 0.6 mg/kg). Intravenous dosing of all patients was completed within 1 minute.

Intraoperative preparation of emergency items, including an anesthesia ventilator, supplemental oxygen, suction tube, a suitable mask, and tracheal tube were conducted according to established protocols. If the SpO_2_ value dropped below 95%, oxygen was delivered using a pressure mask. If body movements occurred during surgery that interfered with the operation, propofol 0.5 mg/kg was administered intravenously while the child’s respiratory and hemodynamic status was continually monitored. After the surgery was complete and the child regained consciousness and exhibited stable vital signs, the parents accompanied the child back to the recovery ward.

### 2.2. Observation indexes and assessment criteria

General conditions during surgery: The anesthesia assistant recorded the duration of anesthesia maintenance during surgery, the duration of surgery, the dose of additional propofol administered intraoperatively, and patient awakening time.

Vital signs: HR, mean arterial pressure (MAP), and SpO_2_ were recorded at T0 (before anesthesia administration), T1 (when the child fell asleep), T2 (when the blood vessel was punctured), and T3 (when the child woke up).

Incidence of intraoperative body movements: The anesthesia assistant recorded the number of intraoperative body movements, which was defined as the number of movements of limbs during surgery that influenced successful completion of the operation by the surgeon.

Incidence of postoperative nausea and vomiting: The anesthesia assistant recorded the number of postoperative incidents of nausea and vomiting.

Incidence of postoperative agitation: The incidence of patient agitation was evaluated after surgery using the Cravero scale. Scores were assessed as follows: a score of 1 indicated sleep and no response to stimuli; a score of 2 indicated sleep with response to stimuli; a score of 3 indicated awake with response to stimuli; a score of 4 indicated difficulty in achieving a state of calm with uncontrollable crying; a score of 5 indicated inability to remain quiet with outbursts of confusion and delirium. Importantly, a score of ≥ 4 for a duration of ≥ 5 minutes indicated the patient experienced awakening period agitation.^[3]^

In this study, no patients were dropped out, such that the final trial population included 60 patients. Additional details pertaining to study patients are presented in the trial flow chart.

### 2.3. Statistical analysis

Trial data were statistically analyzed using SPSS version 17.0 (SPSS, Chicago, IL). Statistical data were expressed as mean ± standard deviation (x̄ ± s). After analyzing the distribution of normality of the data, statistics that obeyed normal distribution were expressed as mean ± standard deviation (x̄ ± s). Comparisons between groups at the same time point and multiple groups comparisons at different time points were analyzed by SNK-q test. Comparisons between two time points within groups were analyzed by matched sample *t* test. Data that did not obey normal distribution were expressed as median (quartiles). Comparisons between groups were performed by rank sum test. The enumeration data were expressed as rate (%), and the chi-square χ^2^ test was used for comparison. *P* values of <.05 were considered to be a statistically significant difference.

## 3. Results

Comparisons of intergroup differences related to general conditions of patients revealed no significant differences among patient groups in anesthesia time and operation time (*P* = .885, *P* = .527, respectively). However, the slightly longer Group H patient waking time was statistically different (*P* = .039) from that of Group L patients, as was the intergroup difference in intraoperative propofol times (*P* = .009), as are shown in Table 1.

**Table 1:** Comparison of general conditions of patients in each group (¯x ± s).

Group	Male/female	Age (yr)	Body mass (kg)	Operation time (min)	Anesthesia time (min)	Additional propofol times (times)	Waking time (min)
Group L (n = 20)	20 (11/9)	3.1 ± 1.8	11.6 ± 5.1	49.7 ± 7.2	54.0 ± 7.8	2.0 (1.0, 2.5)	14.6 ± 2.3
Group M (n = 20)	20 (12/8)	3.8 ± 2.0	12.5 ± 6.3	47.3 ± 10.1	55.5 ± 9.8	1.0 (1.0, 2.0)	16.8 ± 3.3
Group H (n = 20)	20 (9/11)	3.5 ± 1.6	12.1 ± 5.9	48.9 ± 9.0	53.7 ± 8.2	1.0 (1.0, 0)	17.8 ± 2.2^*^
*P* value	.925	.553	.896	.527	.885	.009^†^	.039

Numerical data are expressed as means ± SD. Data that did not obey normal distribution were expressed as median (quartiles).

Compared with group L,

* *P < *.05,

† *P < *.01.

Comparison of intraoperative vital signs of patients in each group:

HR: in Groups L, M, and H, statistically significant differences in HR were observed at time points of T0, T1, T2, and T3 (*P* = .017; *P* = .001; *P* = .005, respectively). In Group L, a statistically significant increase in HR was observed at T2 as compared to that observed at T0 (*P* = .003), while a statistically insignificant increase in HR of 5.88% was observed at T2 as compared to that observed at T1 (*P* = .161). In Group M, statistically significant increases in HR at both T1 and T2 time points as compared to that observed at T0 were observed (*P* = .019; *P* = .003, respectively), while HR increased at T2 by 2.47% as compared to that at T1, although this difference was not statistically significant (*P* = .316). In Group H, HR values increased significantly at both T1 and T2 time points as compared to the HR at T0 (*P* = .012; *P* = .005, respectively), while HR increased at T2 by 0.81% relative to that at T1, although this difference was not statistically significant (*P* = .432). MAP: at all four time points (T0–T3) no statistically significant intergroup differences in MAP values were observed (*P* = .587). SpO_2_: SpO_2_ was significantly increased in all three groups at T1 to T3 time points as compared to that observed at T0, as shown in Table 2.

**Table 2:** Comparison of vital signs in three groups (¯x ± s).

Group	HR (beats/min)			MAP (mmHg)			SpO_2_ (%)		
	Group L	Group M	Group H	Group L	Group M	Group H	Group L	Group M	Group H
T0	111 ± 11	118 ± 10	113 ± 10	66.3 ± 2.1	67.7 ± 2.7	67.8 ± 2.3	97.2 ± 2.0	96.9 ± 2.1	97.0 ± 2.0
T1	119 ± 11†	121 ± 9†	124 ± 9†	64.1 ± 1.5	65.1 ± 1.8	66.6 ± 2.3	99.5 ± 0.5†	99.2 ± 0.9†	99.0 ± 1.0†
T2	126 ± 11†	124 ± 10†	125 ± 8†	69.5 ± 3.2	68.8 ± 2.7	66.1 ± 2.9	99.9 ± 0.3†	99.6 ± 0.7†	99.9 ± 0.3†
T3	115 ± 12*	110 ± 10*	114 ± 9	66.5 ± 2.8	66.6 ± 3.5	68.1 ± 2.2	99.8 ± 0.4†	98.5 ± 1.4†	98.2 ± 0.4†
*P* value	.017	.001	.005	.301	.468	.339	.000	.000	.000

Numerical data are expressed as means ± SD.

HR = heart rate, MAP = mean arterial pressure, SpO_2_ = pulse oxygen saturation, T0 = preoperative, T1 = at the time of anesthesia maintenance, T2 = at the time of arteriovenous puncture, T3 = when the patients awakened.

* *P* < .05, compared with T0.

† *P* < .01, compared with T0.

Occurrences of adverse reactions in each group: Incidence of body movements: the intergroup difference in incidence of intraoperative body movements was statistically significant (*P* = .044). Incidence of agitation during the awakening period: no statistically significant intergroup differences in numbers of incidents of agitation during the awakening period were observed (*P* = .732). Incidence of postoperative nausea and vomiting: no statistically significant intergroup differences in numbers of incidents of postoperative nausea and vomiting were observed (*P* = .887). Incidence rates of adverse reactions are shown in Table 3.

**Table 3:** Adverse reactions of patients in each group (¯x ± s).

Group	Intraoperative body movement rate (%)	Incidence of agitation in awakening period (%)	Incidence of postoperative nausea and vomiting (%)
Group L (n = 20)	35 (7/20)	25 (5/20)	15 (3/20)
Group M (n = 20)	15 (3/20)	20 (4/20)	20 (4/20)
Group H (n = 20)	5 (1/20)^*^	15 (3/20)	20 (4/20)
*P* value	.048	.732	.887

Compared with group L,

* *P* < .05.

## 4. Discussion

Performance of cardiac interventions to treat pediatric CHD patients is becoming clinically common, in spite of challenges associated with maintaining patients in a calm and quiet state during the procedures. For example, if the child cries or is uncooperative, the operation cannot be performed, since a state of calm must be maintained to prevent hemodynamic changes.

Due to specific risks associated with cardiac interventional procedures and with pediatric CHD itself, the incidence rate of adverse events related to pediatric interposition-associated surgical anesthesia is about 0.96%. Common adverse events include respiratory depression, severe arrhythmia, and cardiac arrest.^[4]^ Nevertheless, commonly used propofol intravenous anesthesia is effective, has a rapid onset of action, and is associated with rapid awakening. Notably, administration of propofol in combination with ketamine enhances anesthesia-associated cardiovascular stability that can effectively reduce incidence rates of intraoperative complications, postoperative adverse events, and the probability of occurrence of dangerous events in the catheterization room.^[1]^ Moreover, as compared with ketamine, the induction and maintenance of general anesthesia incorporating S(+)-ketamine requires a smaller anesthesia dose, exerts a stronger analgesic effect, leads to more rapid awakening, less respiratory depression, less secretion, and higher relative anesthetic safety. Thus, use of S(+)-ketamine in combinations with anesthetics such as propofol can provide additional advantages in clinical settings, especially for pediatric surgeries conducted in the catheterization room.^[2]^

Importantly, S(+)-ketamine use leads to increases in salivary and bronchial secretions, which can induce laryngospasm in severe cases, requiring routine use of cholinergic receptor blockers (e.g., atropine) before anesthesia is administered in order to inhibit glandular secretory activities. Although in this study atropine was used preoperatively to elevate the HR in order to avoid propofol-induced HR decrease along with the abovementioned measures to ensure adequate cardiac output, it also inhibited cholinergic receptor activities to reduce glandular secretion of saliva.

Notably, here we observed increased HR in all patient groups after administration of S(+)-ketamine, with the largest increase of 9.78% observed in Group H. This result may be related to the stimulatory effect of S(+)-ketamine in exciting the sympathetic nerve to increase the HR.^[5]^ More importantly, this effect was dose dependent, since Group H received the largest dose of S(+)-ketamine and was the group associated with the most significant HR increase. As compared with HR observed at the T1 time point, the maximum increase in HR (5.8%) was observed at the T3 time point in group L, while the minimum increase in HR (0.81%) was observed in group H, a result that may have been due the use of the lowest dose of S(+)-ketamine in Group L patients that was associated with a weaker analgesic effect. Meanwhile, the most obvious pain-related stimulation occurred when the blood vessel was punctured by the surgeon, as reflected by the maximum increase in HR that was observed at this time point. However, the opposite effect was observed in group H, which reflected a suppressive effect of S(+)-ketamine on pain. Although the HR was fastest in group H, lowest in group L, and intermediate in group M at the T1 time point, intergroup differences were not statistically significant, with lack of significance possibly related to the small sample size of subjects included in this study.

The mechanism of S(+)-ketamine action on cardiovascular system activity is mainly related to its excitation of sympathetic nerve centers and enhancement of sympathetic activities of peripheral blood vessels and thus has relevance to this study. Here, HR and MAP increased in all three groups of patients after intravenous administration of S(+)-ketamine, a result which is consistent with known effects of S(+)-ketamine to markedly increase the HR while also increasing MAP, but to a lesser degree. Here, administration of S(+)-ketamine led to statistically significant HR increases in all three groups that were accompanied by MAP increases that were statistically insignificant. These contradictory results may reflect the fact that blood pressure is affected by several factors, such as HR, vascular contractility, peripheral vascular resistance, blood volume, and heart rhythm, as well by as additional factors that further obscure the overall association between S(+)-ketamine and blood pressure. In addition, in the catheterization room we used a cuff to measure blood pressure every 1.5 min and thus we might not have recorded all blood pressure fluctuations that occurred. Nevertheless, MAP increases that were recorded suggest that the S(+)-ketamine effect in elevating HR exerted a more direct and obvious effect on MAP as compared to MAP-related effects exerted by the other factors. Indeed, the great fluctuations of HR and blood pressure that were observed in patients of the small and medium dose groups during femoral artery puncture may have been related to weaker pain suppression in those patients as compared to patients receiving the highest S(+)-ketamine dose. Thus, this result suggested that a larger dose of S(+)-ketamine was needed to suppress the trauma-related stimulus induced by femoral artery puncture in order to stabilize the patient’s circulatory status, as supported by more stable vital signs observed in patients belonging to this group as compared to patients in the other two groups.

Intriguingly, we observed that the preoperative oxygen saturation was basically normal in all groups. After administration of sedation and analgesia, patients’ oxygen saturation levels significantly improved as compared with the preoperative period, with improved oxygen saturation during surgery likely related to the administration of oxygen via a nasal cannula to patients after they were admitted to the catheterization room. After the surgery in the recovery room, patients remained on oxygen that likely led to higher oxygen saturation at the time of awakening (T3) as compared to that at the time of admission (T0). Nevertheless, oxygen saturation levels were slightly lower than they were intraoperatively, as result probably related to metabolism of the drug as well as to the weakening of respiratory excitation that was associated with surgical pain-related stimulation. Thus, we can hypothesize that none of the three doses of S(+)-ketamine induced respiratory depression and that all doses fell within a clinically safe range. We should mention here that although we monitored patient oxygen saturation during oxygen inhalation throughout the surgery, future studies are needed to assess the effect of S(+)-ketamine on pediatric patient air inhalation function.

Here we should mention that body movements during cardiac intervention surgery differed between groups as an important consideration when choosing appropriate doses of anesthetics. After analgesic sedation was administered to each child, the interventionalist administered a local anesthetic drug before performing the femoral artery puncture. Nevertheless, the sting of the local anesthetic needle and the puncture needle also induced movements of the child’s body. Moreover, insertion of the catheter also had a certain stimulating effect on the heart during the surgical operation, which also caused body movements that affected the operation. Here we observed significantly lower incidence rates of body movements during surgery in patients of the high-dose S(+)-ketamine group as compared to patients in the low-dose group, even though the former group of patients received a lower additional dose of propofol, thus reflecting the stronger analgesic effect of S(+)-ketamine in the high-dose group. Meanwhile, results of a reported study indicate that a even small dose of S(+)-ketamine may be adequate when used to redress a wound in adults.^[6]^ By contrast, here we found that a small dose of S(+)-ketamine could not help our pediatric patients tolerate sterile stimulation, since children cannot mentally cope with the stress of upcoming surgery and thus require greater analgesic sedation. Therefore, the S(+)-ketamine high-dose group of children exhibited the lowest incidence rate of body movements during surgery and were able to fully cooperate with the procedure.

As another important consideration related to anesthesia selection, pediatric general anesthesia awakening agitation is associated with a variety of factors, including young age, separation anxiety due to the absence of parents, adverse postoperative stimuli (including hypoxia, hypothermia, indwelling catheter, urinary retention, pain, etc.), and effects of anesthetic drugs.^[7]^ Wakening agitation occurred in all groups of pediatric patients in this study with similar incidence rates, suggesting that effects of the three S(+)-ketamine doses used here on awakening agitation in our pediatric patients did not differ significantly, warranting further study to confirm these results in clinical trials based on larger numbers of study subjects.

As a final important point, incidence rates of postoperative nausea and vomiting should also be considered when selecting anesthesia drugs. Here we found that postoperative nausea and vomiting incidence rates for the three groups of children were basically the same, with rates peaking at 20%, a lower rate than rates previously reported after pediatric cardiac surgeries (40–80%).^[8]^ Nevertheless, the occurrence of postoperative nausea and vomiting in children is related to surgical method, anesthesia drugs, patient age, preoperative anxiety, mental tension, etc. Here our patients were young children who exhibited anxiety prior to surgery as a factor that may have led to postoperative nausea and vomiting, prompting us to select propofol with S(+)-ketamine for use as intravenous anesthesia, since propofol use can prevent occurrences of these adverse effects in patients. Moreover, propofol use can lead to shorter operation times, a need for fewer types of drugs, and use of simpler procedures. Furthermore, Eich et al^[9]^ showed that when anesthesia for pediatric MRI included a small dose of S(+)-ketamine combined with propofol, the dosage of propofol could be reduced. Notably, use of a lower dose of propofol ultimately reduced the incidence rate of respiratory depression and led to more rapid awakening without increasing the rate of occurrence of side effects in MRI patients who typically do not experience pain from the procedure. However, a small dose of S(+)-ketamine, even when combined with propofol, would likely be inadequate when used to sedate pediatric CHD patients undergoing cardiac interventions, since these patients experience puncture-induced pain and discomfort that require sufficient analgesia and satisfactory sedation to prevent body movements from occurring during the operation. Therefore, we found that medium and large doses of S(+)-ketamine combined with propofol were superior to low-dose S(+)-ketamine when used for sedation of pediatric patients undergoing cardiac interventional surgeries. In this study, we found that the children in the high-dose group had the longest awakening time from anesthesia and the difference was statistically significant compared with the small-dose group. The awakening time of the children in the high-dose group was longer, and we considered that it was related to the higher dose of S(+)-ketamine.But the difference in awakening time between the high-dose and medium-dose groups was not significant. Therefore, we believe that the role of awakening time in this study is not important.This requires us to do more work in the future.

## 5. Conclusion

In this study, S(+)-ketamine dose was varied when administered in combination with propofol as intravenous anesthesia. Due to the small sample of subjects in this study, the results should be used for clinical reference only. In future studies we plan to find a more reasonable dosing regimen by adjusting the doses of both drugs. In the meantime, 0.6 mg/kg S(+)-ketamine combined with propofol provided the best anesthetic effect when administered during cardiac intervention in children with CHD.

## Acknowledgments

The authors would like to express their sincere gratitude to all the staff at the Clinical Investigation Center who were involved in the study.

## Author contributions

**Conceptualization:** Wenjun Liu.

**Data curation:** Fei Xu.

**Formal analysis:** Wenjun Liu.

**Investigation:** Wenjun Liu, Liyun Zhao.

**Methodology:** Wenjun Liu.

**Project administration:** Wenjun Liu, Liyong Guo, Hong Li.

**Supervision:** Jun Ma.

**Validation:** Wenjun Liu.

**Writing – original draft:** Wenjun Liu.
